# Rheological and Electrical Study of a Composite Material Based on an Epoxy Polymer Containing Cyclotriphosphazene

**DOI:** 10.3390/polym12040921

**Published:** 2020-04-16

**Authors:** O. Dagdag, M. El Gouri, A. El Mansouri, A. Outzourhit, A. El Harfi, O. Cherkaoui, A. El Bachiri, O. Hamed, S. Jodeh, G. Hanbali, B. Khalaf

**Affiliations:** 1 Laboratory of Industrial Technologies and Services (LITS), Department of Process Engineering, Height School of Technology, Sidi Mohammed Ben Abdallah University, P.O. Box 2427, Fez 30000, Morocco; gouri_mustapha@yahoo.fr; 2 LPSCM, Department of physics, Faculty of Sciences Semlalia, Cadi Ayyad University, Marrakech PB 2390, Morocco; a.elmansouri@ucam.ac.ma; 3 Nanomaterials for Energy and Environment Laboratory, Cadi Ayyad University, Marrakech PB 2390, Morocco; aoutzour@ucam.ac.ma; 4 Laboratory of Advanced Materials and Process Engineering, Department of Chemistry, Faculty of Science, Ibn Tofail University, BP 133, Kenitra 14000, Morocco; a.elharfi@outlook.fr; 5 Higher School of Textile and Clothing Industries, Laboratory REMTEX, Oulfa BP 7731, Casablanca, Morocco; cherkaoui@esith.ac.ma; 6 University Department, Royal Naval School, Sour Jdid Boulevard, Casablanca B.P 16303, Morocco; abderrahimelbachiri71@gmail.com; 7 Department of Chemistry, An-Najah National University, Nablus P. O. Box 7, Palestine; ohamed@najah.edu (O.H.); g.hanbali@najah.edu (G.H.); bayan.kh107@hotmail.com (B.K.)

**Keywords:** epoxy resin, HGCP, composite, rheological and electrical behavior

## Abstract

In this work, we have studied, formulated, prepared, and characterized the rheological and electrical behavior of a composite material based on an epoxy resin Diglycidyl Ether of Bisphenol A (DGEBA) reinforced with hexaglycidyl cyclotriphosphazene (HGCP). The epoxy system was cured with 4,4’-methylene dianiline (MDA). DGEBA-HGCP-MDA epoxy composite materials with reinforced HGCP which varied from 5% to 10% by weight were prepared by mixing in the molten state. The morphology was evaluated by SEM. The rheological behavior was studied using small deformation rheology. The electrical characterization was carried out with a frequency variation range from 1 Hz to 100 KHz at room temperature. These measurements revealed that the rheological and electrical behaviors strongly depend on the quantity of HGCP in the DGEBA matrix. The linear viscoelastic properties study reveals that the modulus of elasticity G’ is dependent on the amount of HGCP present in the epoxy resin DGEBA. The capacitance-frequency measurements suggest a distribution of localized states in the band gap of the blends.

## 1. Introduction

Epoxy resins are thermosetting polymers that have been extensively employed in various areas such as aerospace, coatings, adhesives, electronic devices, laminates and encapsulations due to the characteristics of outstanding mechanical and electrical properties, relatively low curing shrinkage, superior adhesion to substrates as well as good thermal, chemical and corrosion resistance [1,2,3,4,5,6,7,8]. 

Hexachlorocyclotriphosphazene is a procured oligomer usually used for the synthesis of phosphazene-based polymers [9,10]. The groups like chlorine which are attached to the phosphorus atoms are usually treated as good leaving groups which are easily replaced by various nucleophiles to form cyclotriphosphazene reagents. incorporation of cyclotriphosphazene in a thermosetting polymer network could add several advantages to the polymer like for instance, it enhances the network thermal, mechanical and electrochemical properties. This could be attributed to the synergy of phosphorus and nitrogen [11,12,13]. However, they have poor compatibility with epoxy resin which leads to reduce mechanical properties of composites and limit their application. This problem might be solved by the technique of chemical modification. By grafting the organic group on cyclotriphosphazene, the compatibility is supported between the inorganic layer of cyclotriphosphazene and organic layer of the epoxy resin matrix. This technique is similar with that concerning the enveloping microscopic amounts of matter (solid particles, droplets of liquids, or gas bubbles) in a thin film of polymer which forms a solid wall [14,15]. This core/shell structure allows the isolation of the encapsulated substance from the immediate surroundings and thus protects it from any degrading factors such as temperature and improves the dispersion.

In this work the three epoxy components (DGEBA-HGCP MDA) was reinforced by various amount of hexaglycidyl of cyclotriphosphazene (HGCP). The amounts of HGCP ranged from 5% to 10%. The epoxy system was cured with the MDA. Produced thermosetting network was characterized by various techniques that include morphology by SEM, rheology by HAAKE MARS Modular Advanced Rheometer System and electrical study by Keithley 3330LCZ impedance meter.

## 2. Experimental

### 2.1. Materials and Methods

**Figure 1:**
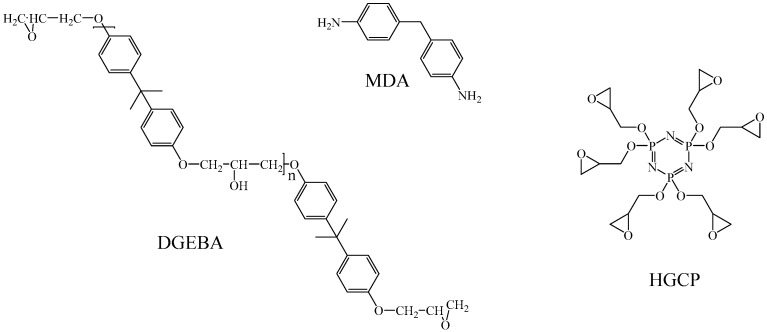
Chemical structures of the epoxy materials and the curing agent.

The chemical structures of the materials studied in this work are represented in Figure 1. The material was diglycidyl ether of bisphenol A (DGEBA) type Epon 828, the curing agent 4,4′-methylene dianiline (MDA) and hexaglycidyl of cyclotriphosphazene (HGCP). The synthesis of HGCP resin was carried out according to a published procedure [16].

### 2.2. Sample Preparation

**Figure 2:**
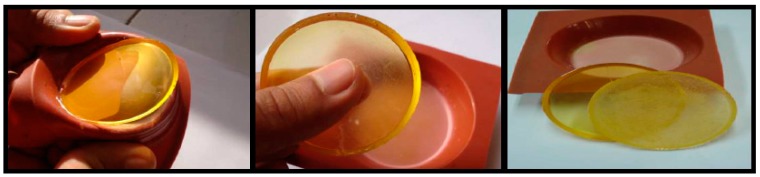
Sample preparation technique.

Mixing the polyepoxide and the hardener (curing agent) produced a bridged three-dimensional network as shown in Figure 2. The curing process was exothermic, the crosslinked materials were hard. They could therefore respond to use in a wide temperature range. The procedures of mixing epoxy resin with hardener before crosslinking was followed by Levin [17]. MDA was crystallized at room temperature, then placed in an oven at 120 °C (more than melting point) and the resin was brought to 70 °C. After melting, the was mixed to form a single phase which was at 70 °C. The samples thus prepared were sealed in Teflon molds and underwent the following cooking cycle:

- One night at 70 °C
- 3 h at 100 °C
- 2 h at 120 °C
- 1 h at 140 °C
- 30 min at 150 °C

### 2.3. HGCP Dispersion Approach in a Polymer Matrix

**Figure 3:**
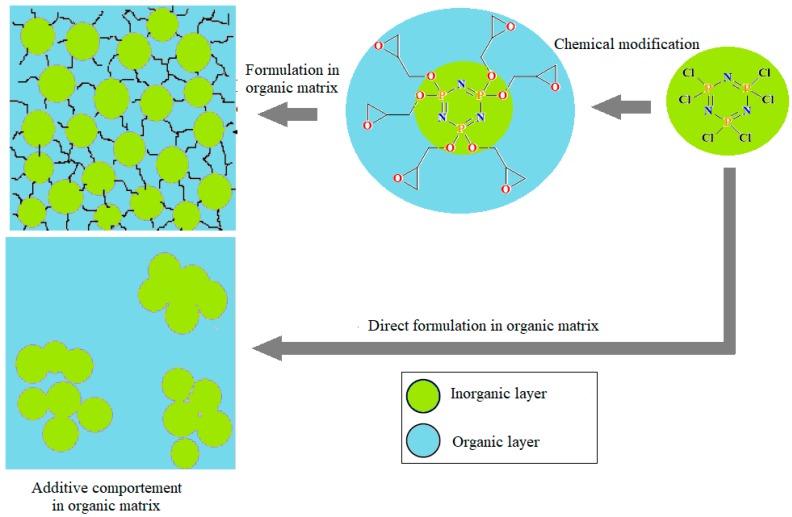
Dispersed hexaglycidyl of cyclotriphosphazene (HGCP) in an organic matrix diglycidyl ether of bisphenol A (DGEBA).

Numerous studies have already demonstrated that the physical properties largely depend on the degree of dispersion of the nanoparticles in the polymer matrices. Mainly, the influence of the degree of dispersion on the rheological and mechanical properties is well known. The absence of chemical bonds and compatibility between the polymer and the particle, which has a link with the dispersion, can influence the mechanical properties of the composites because a strong interfacial bond can effectively transfer the load from the matrix to the reinforcement. Often used are the chemical surface modification of the inorganic nanoparticle, the modification of the deposition reaction, the chemical modification of mechanical force, the modification by high energy and the modification by polymer surface grafting and intercalation. These changes resolve the heterogeneous aggregation issues. The interactions between the reactive groups of the polymer and the nanoparticles depend on the chemical structure of the polymer and the surface charge of the nanoparticles. Good dispersion was expected due to the organic group on the HGCP and since DGEBA is epoxy resin. Due to the compatibility between two species, the flame retardant additive dispersed in the DGEBA resin and no agglomerate was observed (Figure 3) [18].

Compared to the composite ([NPCl_2_]_3_-DGEBA), the aggregations were observed in the latter composite. This suggests that the modified chlorocyclophosphazene may be well dispersed in DGEBA, which resulted from improved compatibility with DGEBA resin and the crosslinked structure of cyclophosphazene. In this work, while HGCP had good dispersion in DGEBA epoxy resin, the study will focus on the effect of HGCP in the physical behavior of materials based on DGEBA epoxy resin.

### 2.4. Morphological Characterization

The observations morphological were carried out on a scanning electron microscope (SEM) of the JEOL-JSM-5500 type (Dearborn, MI, US), whose maximum resolution is 3.5 nm with a magnification ranging from 18 to 300,000 times and automatic focus adjustment (contrast, brightness, stigmatism), the acceleration voltage varies from 0.5 to 30 KV. A computer control system allowed images to be digitized using image processing software (JEOL, Dearborn, MI, US).

### 2.5. Rheological Measurement

For the measurement of rheological properties, an imposed constraint Rheometer of the HAAKE MARS Modular Advanced Rheometer System type (Thermo-Scientific, Karlsruhe, Germany) was used. This instrument is equipped with different measuring bodies. Our study is carried out with a plane-plane geometry with a diameter of 25 mm and a thickness of 1.8 mm. The dynamic oscillatory measurements were made using a frequency sweep ranging from 0.1 to 100 rad/s with a constant applied strain of 0.1% which is in the linear viscoelastic region. All measurements were taken at a constant temperature of 100 °C.

### 2.6. Electrical Measurements

The electrical measurements were carried out at room temperature on samples. The impedance measurements (capacitance-frequency) were carried out using a Keithley 3330LCZ impedance meter (Oceanside, CA, US). All instruments are controlled by a computer via a GPIB card.

## 3. Results and Discussions

### 3.1. Morphology Study

**Figure 4:**
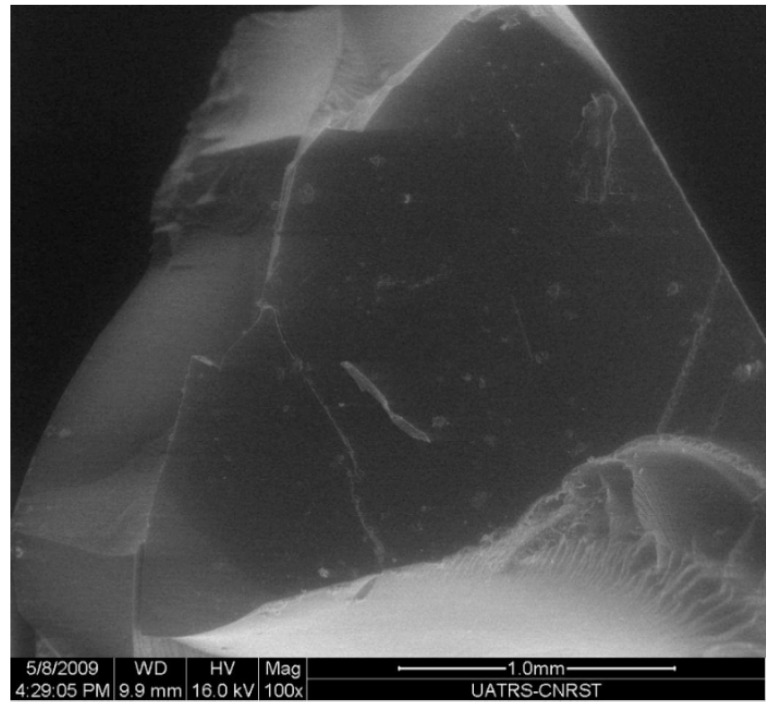
SEM micrograph of the DGEBA samples mixed with HGCP.

The SEM micrograph of DGEBA sample mixed with HGCP is shown in Figure 4. For all quantities of 10% HGCP, the sample was uniform and examination of their surface morphology revealed no pinholes or porosity.

### 3.2. Dynamic Viscoelastic Analyzes

**Figure 5:**
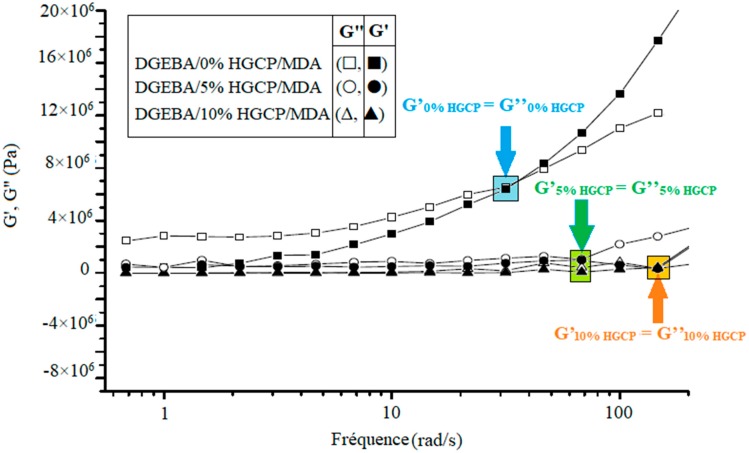
Modulus of elasticity G’ and modulus of loss G” as a function of the frequency of the composites.

Work on the rheological behavior of composites, i.e., macroscopic dynamic viscoelastic behavior, has often made it possible to better understand the structures and relationships responsible for the reinforcement of polymers by HGCP particles. Rheology therefore places itself here on the border between structural determination and physical properties of composites; moreover, this technique is often the key in understanding and improving the implementation. The elastic modulus G’ (solid symbols) and the loss modulus G “(open symbols) of the DGEBA / HGCP / MDA composites were measured in the linear range at 100 °C and in a frequency range from 0.1–100 rad/s, the results are given in the Figure 5.

These measurements were obtained by applying a constant deformation of 0.1%, which is in the linear viscoelastic region. The results show that all the 0%, 5% and 10% mixtures of HGCP dispersion behaved like a solid, with G ‘and G “almost independent of frequency and G’> G”. The value of G’ and G “decreased with increasing amount of HGCP in the epoxy resin DGEBA. At high frequency, we can observe the crossover frequency when G’ = G”. The latter increased with the increase in the quantity of HGCP, which reflects the elastic behavior of the samples. These effects were also observed on other polymers [19]. These composites would be caused by long distance density fluctuations, kinetically frozen at measurement frequencies due to their extremely long relaxation [20]. Furthermore, the increase in the storage module and the loss module for different composites is a common phenomenon for the crosslinked and reinforced epoxy resin, which can be explained by the interaction between the resin and the HGCP hindering the movement of the macromolecular chains [21,22].

**Figure 6:**
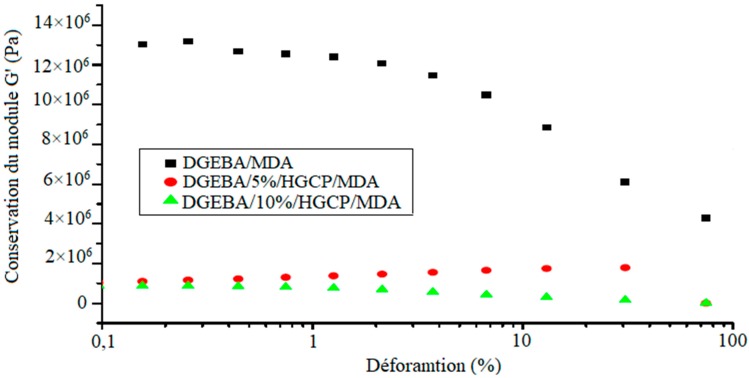
Conservation of the module G’ as a function of the deformation for the DGEBA/HGCP/MDA samples.

Figure 6 shows the conservation of the module G′ as a function of the deformation for the DGEBA samples with different quantities of HGCP (0%, 5% and 10%).

In all cases, an increase in the linear field was observed when the amount of HGCP increased. For these systems where the HGCP was well dispersed in the DGEBA, the curves did not show any particular characteristic compared to the linear field for a pure DGEBA.

**Figure 7:**
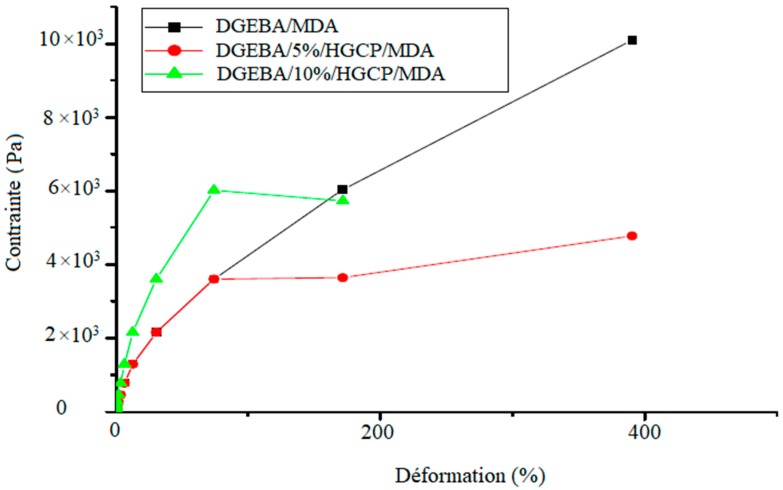
Stress-strain curves for DGEBA samples with different amounts of HGCP (0%, 5% and 10%).

The stress-strain curves for the DGEBA samples with different quantities of HGCP (0%, 5% and 10%) are illustrated in the Figure 7.

The mechanical properties of the DGEBA material modified by the addition of HGCP exhibited better ductility compared to pure DGEBA-MDA and also he observed that the breaking strain of the DGEBA-HGCCP-MDA material was significantly higher than that of the DGEBA resin pure. The presence of HGCP in the DGEBA resin results in the appearance of a plastic deformation zone from 74.33% deformation. However, the tensile strength decreased after the introduction of 10% HGCP into the DGEBA resin and the modulus of elasticity by a factor of 2 which can be attributed to the increase in knots in the matrix. In other cases, the result indicates that the introduction of HGCP into the DGEBA resin makes the samples more flexible. This increased flexibility of the samples can be attributed to the free volume presented by the HGCP / MDA / DGEBA network, promote molecular movement and improve the tensile strength [23].

### 3.3. Electric Properties

**Figure 8:**
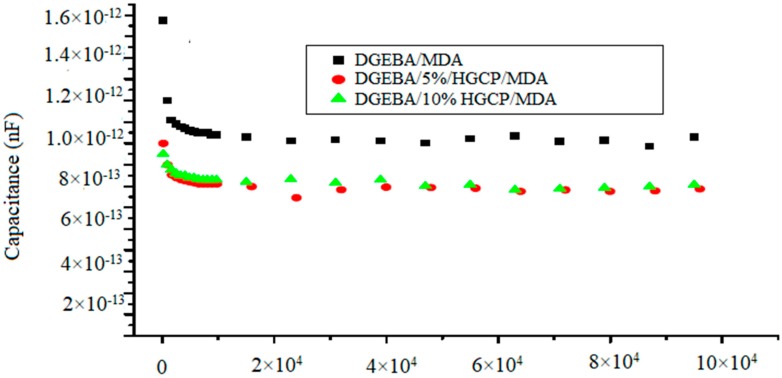
Capacity-frequency characteristics for DGEBA samples with different quantities of HGCP (0%, 5% and 10%).

Figure 8 shows the capacity-frequency characteristics of (DGEBA/HGCP/MDA) in the frequency range 1–100 kHz. The capacity decreased by a factor of two when the frequency was increased to 100 kHz. This dispersion of capacity can be explained by the distribution of the localized states in the band gaps of HGCP network [24], barrier inhomogeneities and series resistance. At high frequency (1 kHz), the deep states could not follow the barrier modulation and the capacitance must be replaced by the dielectric capacitance of the matrix. In addition, for 5% and 10% of the quantity of HGCP, the capacity of the samples increased. This could be due to the increase in nods in the samples.

## 4. Conclusions

Various composite materials based on the epoxy resin DGEBA, MDA as hardener and the epoxy resin HGCP as an additive were prepared and formulated. The presence of a small amount (5% and 10% by weight) of HGCP showed high improvement in the mechanical and electrical properties of the DGEBA based epoxy materials. The chemical modification of cyclophosphazene facilitated better dispersion by optimizing the compatibility between the organic matrix and the cyclophosphazene. The presence of HGCP in the DGEBA resin showed a significant improvement in flexural modulus and hardness, which could be attributed to the flexibility of HGCP within a matrix stretched via the free volume present in the DGEBA network and also appearance of a plastic deformation zone from 74.33% deformation. However, the tensile strength decreased after the introduction of 10% HGCP into the DGEBA resin and the modulus of elasticity by a factor of 2. In addition, for 5% and 10% of the quantity of HGCP in the DGEBA, the capacity of the samples increases. This could be due to the increase in nods in the samples.
